# Re-evaluation of the mechanisms of dietary fibre and implications for macronutrient bioaccessibility, digestion and postprandial metabolism

**DOI:** 10.1017/S0007114516002610

**Published:** 2016-07-07

**Authors:** Myriam M.-L. Grundy, Cathrina H. Edwards, Alan R. Mackie, Michael J. Gidley, Peter J. Butterworth, Peter R. Ellis

**Affiliations:** 1 Biopolymers Group, Diabetes and Nutritional Sciences Division, King’s College London, Franklin-Wilkins Building, 150 Stamford Street, London SE1 9NH, UK; 2 Institute of Food Research, Norwich Research Park, Colney, Norwich NR4 7UA, UK; 3 ARC Centre of Excellence in Plant Cell Walls, Centre for Nutrition and Food Sciences, Queensland Alliance for Agriculture and Food Innovation, The University of Queensland, Brisbane 4072, Qsd, Australia

**Keywords:** Plant cell walls, Dietary fibre, Food structure, Bioaccessibility, Gastrointestinal functions

## Abstract

The positive effects of dietary fibre on health are now widely recognised; however, our understanding of the mechanisms involved in producing such benefits remains unclear. There are even uncertainties about how dietary fibre in plant foods should be defined and analysed. This review attempts to clarify the confusion regarding the mechanisms of action of dietary fibre and deals with current knowledge on the wide variety of dietary fibre materials, comprising mainly of NSP that are not digested by enzymes of the gastrointestinal (GI) tract. These non-digestible materials range from intact cell walls of plant tissues to individual polysaccharide solutions often used in mechanistic studies. We discuss how the structure and properties of fibre are affected during food processing and how this can impact on nutrient digestibility. Dietary fibre can have multiple effects on GI function, including GI transit time and increased digesta viscosity, thereby affecting flow and mixing behaviour. Moreover, cell wall encapsulation influences macronutrient digestibility through limited access to digestive enzymes and/or substrate and product release. Moreover, encapsulation of starch can limit the extent of gelatinisation during hydrothermal processing of plant foods. Emphasis is placed on the effects of diverse forms of fibre on rates and extents of starch and lipid digestion, and how it is important that a better understanding of such interactions with respect to the physiology and biochemistry of digestion is needed. In conclusion, we point to areas of further investigation that are expected to contribute to realisation of the full potential of dietary fibre on health and well-being of humans.

The traditional methodological approach adopted by nutritionists, dietitians and epidemiologists for evaluating the nutritional properties of foods and diets and their impact on human health is largely based on the chemical analysis of food composition. However, this approach alone is inadequate and additional factors such as the structure and properties of foods have to be taken into consideration when studying, for example, the complex, heterogeneous tissues of plant foods. In particular, it is now well known that the physico-chemical properties of dietary fibre are of paramount importance in influencing gastrointestinal (GI) function, notably nutrient bioaccessibility and digestion, microbial fermentation, GI hormone signalling, metabolisable energy and postprandial metabolism^(^ ^1^ ^–^ ^3^ ^)^.

Despite the plethora of literature published on dietary fibre, there is still considerable confusion and disagreement about its definition and how this complex material should be analysed. On the basis of a current physiological definition^(^ ^4^ ^)^, dietary fibre is a generic term that includes carbohydrate-based plant materials that are not digested by endogenous enzymes in the upper GI tract. The main components of fibre are plant cell wall polysaccharides, but this definition also encompasses other non-digestible carbohydrates such as resistant starch and oligosaccharides (e.g. fructans).

Plant cell walls are supramolecular matrices of cellulose, hemicelluloses, pectic substances, non-carbohydrate components (e.g. lignin and protein) and water. The amounts and relative proportions of these components vary depending on the botanical source as well as the type, function and maturity of plant tissue. The heterogeneity in composition and the structure of cell walls explain the wide variation in the properties of the cell wall matrix and its individual polysaccharide constituents (e.g. porosity, cell separation/rupture and viscosity)^(^ ^5^ ^)^. These properties are strongly linked to the physiological impact of fibre on digestion and gut function, including, for instance, effects on nutrient bioaccessibility and rate of gastric emptying/GI transit, inhibition of the flow and mixing efficiency of digesta, changes in the rate and extent of macronutrient digestion/absorption, and prebiotic effects on gut microflora. The role of fibre in physically encapsulating/entrapping nutrients in particular has been identified as a major mechanism by which structurally intact plant tissues tend to be digested at a slower rate and to a lesser extent, thereby attenuating the postprandial rise in glycaemia and/or lipaemia^(^ ^6^ ^–^ ^8^ ^)^. These physiological changes are considered to be of benefit in the dietary treatment and risk reduction of cardiometabolic diseases such as type 2 diabetes and CVD and may also have a positive impact on obesity management^(^ ^9^ ^,^ ^10^ ^)^.

The worldwide emergence of cardiometabolic diseases that are dietary related indicates an urgent need to develop new ingredients and foods with enhanced nutritional benefits. In many Western populations, diets are still often low in dietary fibre, because of the relatively low intake of edible plant tissues from fruits, vegetables and wholegrain cereal products^(^ ^11^ ^,^ ^12^ ^)^. However, not all types of dietary fibre have the same benefits on gut function and metabolism and even the same source of fibre may elicit wide variations in physiological behaviour. A notable example of this is the variations in biological activity of the mixed-linkage polysaccharide (1–3,1–4)-*β*-d-glucan, which is found in the cell walls of oats and other cereals. This soluble form of dietary fibre is considered to be the main component responsible for the property of many oat products to lower fasting blood cholesterol concentrations and postprandial glycaemia^(^ ^13^ ^)^. Variations in the amount and molecular weight of oat *β*-glucan that solubilises in the upper GI tract, which is known to influence intra-luminal viscosity, may explain the marked differences in physiological and clinical efficacy of this polysaccharide. Moreover, a more recent study has highlighted the importance of the physical state of fibre (e.g. the structural integrity of cell walls) in determining the effects of plant foods on physiological functions such as nutrient bioaccessibility and digestion kinetics, a crucial factor of which is the degree of cell wall encapsulation^(^ ^7^ ^,^ ^14^ ^–^ ^16^ ^)^.

A wide range of *in vitro* and *in vivo* methods has been developed and used to investigate the digestion processes, thus providing some insight on the interactions between plant food structure and gut function, and therefore the capacity to predict effects on postprandial metabolism^(^ ^6^ ^,^ ^17^ ^–^ ^25^ ^)^. However, although the metabolic and health effects associated with dietary fibre consumption have been extensively investigated in human intervention studies, the mechanisms that explain these observed effects are far from being fully understood^(^ ^2^ ^,^ ^3^ ^,^ ^26^ ^)^.

This article reviews the current knowledge relating to the structure and properties of plant foods and the mechanisms by which macronutrients especially starch and lipid are released and digested, with a particular focus on the role of dietary fibre.

## Food matrix and nutrient bioaccessibility

### Definitions

**Fig. 1:**
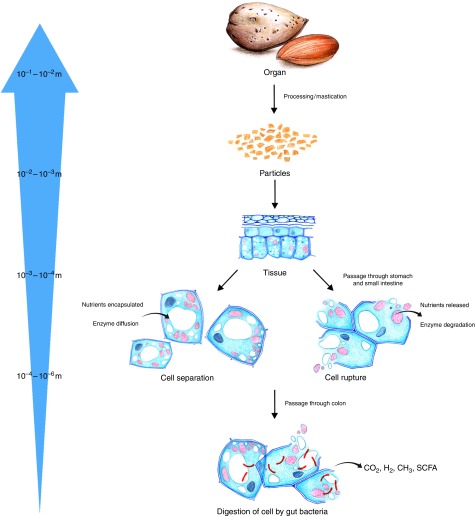
Characteristic multiscale features of plant food from mm dimensions of the plant organ (e.g. almond seeds) to nm scale of intra-cellular contents. Note that the illustrations depicting the structure of tissues and cells are not an accurate representation of almond cells (see Fig. 2. for photomicrographs of almond cells).

The term food matrix describes the physical form of a food, and encompasses the natural structures of raw plant materials as well as the composite organisation that results from industrial and/or household processing^(^ ^27^ ^,^ ^28^ ^)^. For edible plants, the scales range from the cm scale of plant tissues to the nm dimensions of nutrients and phytochemicals inside plant cells (Fig. 1)^(^ ^29^ ^)^.

The physico-chemical attributes of a food matrix can affect the efficiency of the physical and biochemical processes of digestion^(^ ^30^ ^)^. In order for the macronutrients contained in a food to be digested, they need to be in contact with the digestive secretions (i.e. enzymes) – for example, in plant tissues, this could occur either by rupture of the cell walls and release of nutrients into the extracellular environment or by diffusion of the enzymes through a permeable cell wall. However, not all cell wall matrices or individual cell wall polysaccharides in plant foods behave in a similar manner during digestion. Thus, macronutrients of plant foods containing cell walls that are highly permeable or prone to physical disruption *in vivo* (e.g. mastication) will be released (bioaccessible) and/or digested at early stages of digestion. When cell walls are less permeable or less susceptible to rupture, however, there is likely to be a reduction in the rate and extent of nutrient release and digestion. In addition, domestic and industrial processing of plant ingredients and foods, such as hydrothermal treatment (cooking) and milling, can affect bioaccessibility and digestion by modifying the structural integrity of the plant tissue, particularly the cell walls (e.g. cell wall damage and increased porosity and water solubility of cell wall polysaccharides). In addition to these effects, processing can significantly alter the structure and properties of the intra-cellular macronutrients surrounded by the cell wall matrix. For instance, the degree of gelatinisation and/or retrogradation of starch, the extent of protein unfolding and aggregation, the physical state of lipids (e.g. the size of the emulsion droplets) and the quality of the lipid–water interface will all impact on the digestion kinetics of plant foods^(^ ^31^ ^–^ ^34^ ^)^.

Bioaccessibility refers to the proportion of a nutrient or any other substance (i.e. phytochemicals) that is released from the food matrix and is potentially available for absorption in the small intestine^(^ ^27^ ^)^. This term differs from the definition of bioavailability, which incorporates the absorption, metabolism, tissue distribution and biological action of a specific nutrient^(^ ^35^ ^)^. The definition of bioaccessibility may also include nutrients that are still enclosed within the cell but are available to digestive enzymes, as in the case of plant food tissues with permeable cell walls such as durum wheat^(^ ^36^ ^)^.

Bioaccessibility is an important concept that needs to be considered when giving nutritional advice or for designing food products to address specific nutritional requirements. Individuals aiming to reduce their energy consumption would be interested in foods with decreased macronutrient digestion and absorption. On the other hand, for individuals suffering from malnutrition or having higher energy requirements, including, for instance, athletes, the elderly and patients with diseases such as cancer and HIV, it is recommended that they consume nutrient-rich food with high bioaccessibility. In all cases, a full understanding on how the food matrix behaves within the GI tract during digestion and how this affects nutrient bioaccessibility is essential.

**Fig. 2:**
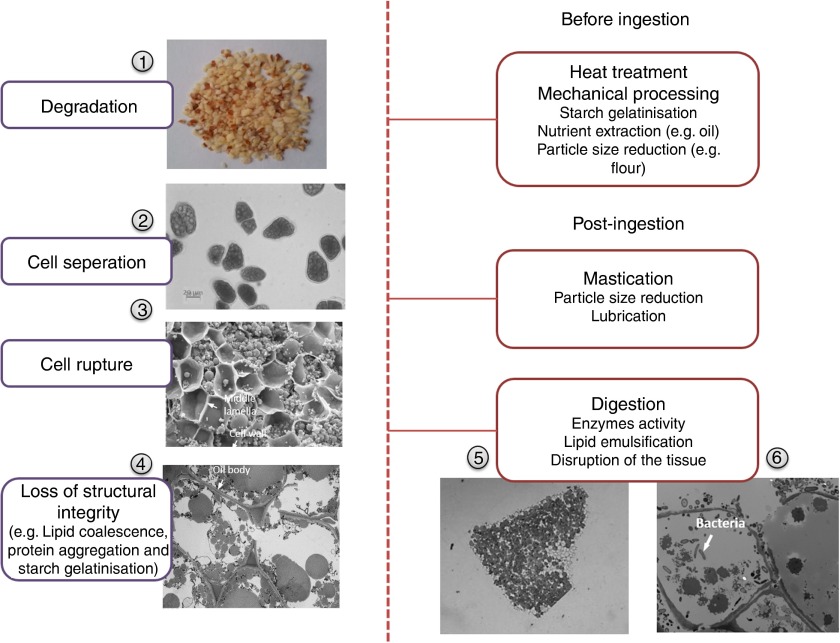
Structural changes in a model food (almond) when processed and/or digested. (1) Ground almond particles of 1 to 2 mm; (2) light microscopy (LM) image of separated almond cells; note that these cells do not exist naturally and are isolated following treatment with cyclohexanediamine tetraacetic acid (CDTA); (3) scanning electron micrograph of the surface of a masticated almond particle; the cells appear to be ruptured but some of their content is still present; (4) transmission electron micrograph (TEM) of fractured almond cells shows damaged cell walls and coalesced lipid; (5) LM image of a digested almond particle (about 1 mm) that has been recovered at the terminal ileum from an ileostomy volunteer; the cells located at the surface of the particles are mainly empty but the majority of the cells still contain nutrients; (6) TEM section of almond tissue from faecal samples shows numerous bacteria that have digested the cell walls and cell content. Note that almond seeds do not contain starch, so starch gelatinisation caused by hydrothermal processing is only relevant to other plant tissues containing starch.

In order to elucidate the role of plant food structure in regulating nutrient bioaccessibility, macronutrients such as lipids and starch may be considered as part of a structural hierarchy (Fig. 1), in which components at the molecular level (i.e. biopolymers) are the building blocks that provide mechanical strength and confer the physico-chemical properties of higher-order structures. A comparison of not only the nutrient composition but also the way nutrients are assembled in plant cells, which make up tissues and organs, provides insight into how different plant materials are likely to be disassembled during food processing and digestion (Fig. 2). For this purpose, plant storage organs represent the highest level of structure described, and typically provide nutrient-rich foods that are grown and harvested for human consumption, including leguminous seeds, cereal grains, tubers, modified stems (e.g. potatoes) and tree nuts.

### Plant food digestion and effect of tissue structure

**Fig. 3:**
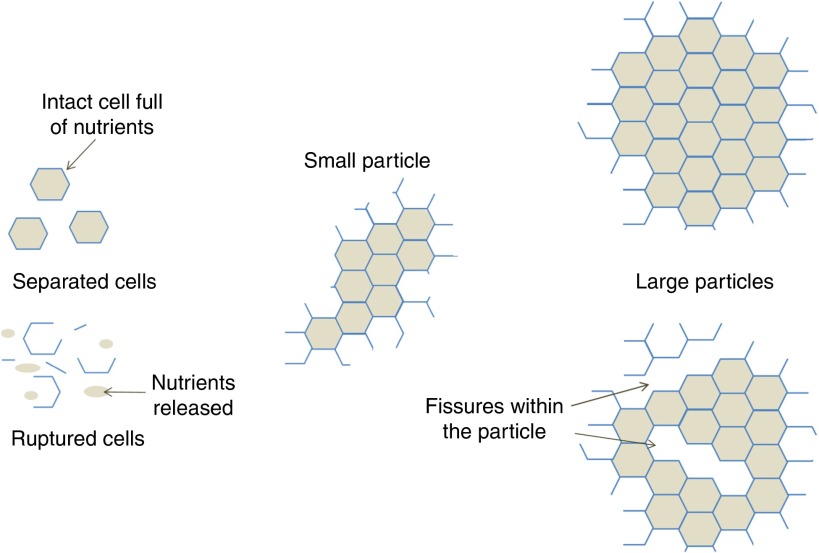
Schematic representation of plant tissue after mastication or mechanical processing.

Mastication is the first stage of the digestion process and consists of breaking down the plant food ingested into smaller particles as well as lubricating it with saliva in order to facilitate its progression through the oesophagus^(^ ^37^ ^)^. During mastication, the food matrix becomes greatly transformed with an increase in surface area and formation of a bolus. The digestion of bioaccessible, cooked starch by salivary amylase leads to rapid reductions in viscosity^(^ ^38^ ^)^. When subjected to mastication, cells within a plant food tissue can either rupture or separate depending on the strength of inter-cellular adhesions^(^ ^1^ ^)^. For example, the cells of cooked (hydrothermally processed) legumes tend to separate, whereas the cells of raw, hard food structures such as nuts usually rupture^(^ ^1^ ^,^ ^14^ ^–^ ^16^ ^,^ ^31^ ^)^. In the primary walls of most dicots and some monocots, the adhesion properties are largely determined by the structure of the pectic polysaccharides and the Ca cross-linking between these polymers in the middle lamella^(^ ^1^ ^)^. The rupture of cells during mastication increases the area of ‘fractured surfaces’ (Fig. 2). The proportion of ruptured cells of hard food materials such as seeds and raw vegetables depends on the number of fractured surfaces created by mastication. Thus, masticated particles of smaller size possess a larger proportion of fractured cells, and therefore exhibit greater nutrient losses (Fig. 3). Furthermore, fissures running through the core of plant tissue particles can be created during mastication, as observed in almonds^(^ ^39^ ^)^. These internal fissures could presumably facilitate the digestion of the nutrients contained within the tissue by enabling the diffusion of digestive agents ‘inside’ the particle and/or the ‘leaching’ out of intra-cellular nutrients, which may or may not have been digested. In contrast, mastication of soft tissues, such as mango, is likely to form particles made of compacted cells that are held together by connective vascular fibres, thus preventing the release of nutrients (carotenoids) from the cells in the oral cavity^(^ ^40^ ^)^.

The size of masticated particles varies greatly between food boluses, with particle dimensions ranging from 5 μm to 3 cm^(^ ^41^ ^,^ ^42^ ^)^. However, a degree of similarity was found among plant food categories: nuts, for instance, tend to have smaller particles than vegetables such as cauliflower, radish and carrot. For boluses formed from hard, brittle foods such as almonds, the inter-individual variability in particle size distribution is small, even though individuals can have different mastication strategies^(^ ^39^ ^,^ ^42^ ^,^ ^43^ ^)^. However, for certain other plant food boluses, for example, cooked rice (i.e. boiled in water), the particle size distribution differs greatly between individuals^(^ ^44^ ^)^. The bolus particle size distribution, which reflects the extent of deformation and disintegration of a plant food, is an important parameter, as it affects the subsequent digestion processes including gastric emptying and sieving^(^ ^43^ ^)^ and nutrient digestibility in the small intestine^(^ ^7^ ^,^ ^16^ ^)^. The extent to which these masticated foods disintegrate depends on many factors including the textural characteristics of the plant tissue, the amount, composition and supramolecular structure of the cell walls, and the physico-chemical properties of the intra-cellular contents^(^ ^1^ ^)^.

Once masticated, plant foods such as nuts and raw vegetables are swallowed and enter the stomach via the oesophagus. In the antrum of the stomach, the particles of food may be further eroded, increasing the available surface area. The stomach acts as a short-term storage reservoir, and thus controls the delivery of chyme to the duodenum. Simultaneous chemical and mechanical processes facilitate further breakdown of food. For digestion to occur in the stomach, the food ingested should therefore be transformed into particles that have a surface that allows the penetration of the endogenous compounds essential for digestion, such as enzymes (i.e. pepsin and gastric lipase) and acids^(^ ^45^ ^)^.

The mode of disintegration of solid foods has been examined recently by Kong & Singh^(^ ^46^ ^)^ who reported that the initial food texture and the changes occurring during mastication and gastric digestion varied greatly among different foods. For instance, these authors suggested that in the stomach, erosion was the main mechanism responsible for the disintegration of nuts. Furthermore, compared with other foods (carrot and ham), raw almonds seem to absorb the highest amount of water in static soaking tests (approximately a 9-fold increase after soaking) and, in a stomach model, show a significant reduction in hardness, as measured using a penetration test on a Texture Analyzer (TA-XT2, Texture Technologies Corp., Scarsdale, NY/Stable Micro Systems). Kong & Singh^(^ ^47^ ^)^ have also suggested that almonds disintegrate over time with a delayed-sigmoidal profile due to water absorption and softening. Therefore, after a prolonged residence time in the aqueous environment of the stomach and the duodenum, the texture of the almond would be modified as well as its mode of disintegration.

Chyme is a heterogeneous mass, containing both liquid and solid materials, including particles of various sizes. It is currently assumed that particles in the stomach need to reach a size <1–2 mm before being emptied into the duodenum^(^ ^48^ ^)^. However, if the particles are too difficult to break down (‘indigestible’), they may eventually flow from the stomach when the emptying of the digestible solid is completed and the fasting motility pattern is resumed^(^ ^49^ ^)^. Considering the cell dimensions of some common edible plant tissues, a 2-mm particle would be expected to contain a high proportion of intact plant cells. For instance, an almond tissue particle of that size (as a cube) consists of approximately 327 000 lipid-rich parenchyma cells of which approximately 19 000 are ruptured surface cells^(^ ^50^ ^)^. Following similar mathematical and geometric principles, and assuming a cell size of 250×50×50 μm, a 2-mm particle of wheat endosperm tissue is estimated to contain approximately 10 000 starch-rich cells of which approximately 3800 are ruptured^(^ ^14^ ^)^. This implies that approximately 62 % of the starch in this particle would be encapsulated within intact plant cells, whereas the remaining 38 % of starch would be exposed and potentially available for digestion on the fractured particle surface.

The nature of food will therefore have an effect on gastric sieving, gastric volume and emptying of the stomach. Some fibre-rich foods have been noticed to influence gastric function by increasing bulk viscosity, which affects gastric sieving, emptying rates, retropulsion and also alters the size of particles in the chyme^(^ ^51^ ^–^ ^54^ ^)^. The proportion of liquid and solid in the meal affects the time required to digest it. Food particles that are large in size, denser and/or harder are known to delay gastric emptying^(^ ^48^ ^,^ ^53^ ^)^. A sieving mechanism takes place in the stomach where liquids and small particles move relatively rapidly through the stomach (during digestive motility), whereas larger and heavier debris are collected in the antrum (during the fasting motility), with free lipids forming a floating layer on the surface of the digesta^(^ ^55^ ^)^. Retropulsion, which corresponds to the ‘return’ of the chyme into the stomach after having been propulsed and ground in the distal stomach area, also occurs.

The main function of the small intestine is digestion and absorption of nutrients, mostly occurring in the upper portion of the small intestine (duodenum and jejunum). To fulfil this role, intestinal enzymes, bile and various electrolytes (particularly Na, Cl, Ca and K) are required. The absorption of nutrients is maximised by the large absorptive area of the intestine. Its inner wall, or mucosa, is folded, with each fold being covered with villi, which in turn are lined with microvilli. The chyme that reaches the small intestine undergoes a process of longitudinal mixing and breaking down by peristalsis and segmentation. The extent to which the chyme is mixed and propelled along the intestinal tract is determined by its rheological properties (e.g. viscosity)^(^ ^56^ ^)^. These properties therefore affect nutrient release and absorption. The digesta is a suspension of particulates, the composition of which changes as they progressively transit along the GI tract. As a result of the secretion of GI juices and the digestion and absorption of nutrients and water, the concentration, shape and size of the particulates are altered, thereby affecting the physico-chemical properties of the digesta.

Plant foods with a hard texture, such as almonds, can resist the shear forces created by peristalsis in the stomach and the small intestine as demonstrated by the large particles remaining after digestion^(^ ^57^ ^)^. At the cellular level, the composition and overall structure of the cell wall matrix, specific to the source of the plant food studied, have consequences on cell wall behaviour in the GI tract and the bioaccessibility of nutrients. During mastication, for example, raw almond cells are known to rupture^(^ ^39^ ^)^, whereas cooked chickpea cells are more likely to separate^(^ ^36^ ^)^. When the cells fracture, their contents become exposed to digestive fluids in the GI tract lumen; macronutrients are digested and the products of digestion are absorbed and may evoke a physiological response (i.e. hormone secretions and their subsequent effects such as adjustment of gastric emptying rate and GI contractions). In contrast, when the cells are intact (separated) and encapsulate the nutrients they contain, they continue to move towards the colon without being further digested. This behaviour was observed in human ileal effluent recovered 3 h after ingestion of white beans^(^ ^23^ ^)^. In addition, early *in vitro* studies have shown that the presence of intact starch-containing cells in leguminous tissues is strongly linked to a relatively low rate and extent of starch digestion^(^ ^58^ ^–^ ^60^ ^)^. Furthermore, in another study, the inclusion of undamaged legume cells in mixed meals was reported to lower significantly the postprandial incremental blood glucose and insulin responses in patients with type 2 diabetes in comparison with the inclusion of damaged legume cells^(^ ^61^ ^)^. Thus, it seems that starch encapsulated within leguminous cells is protected from digestion in the small intestine and produces a low glycaemic response compared with non-encapsulated starch.

In the ileum, the presence of nutrients, particularly lipids, activates the ileal brake – a series of negative-feedback mechanisms that seem to be linked to a reduced food intake by inhibiting gastric emptying and intestinal motility^(^ ^62^ ^)^. This process results in ‘optimising’ the digestion of nutrients by slowing down the physiological activity of GI motility in the upper parts of the GI tract, making the main site of absorption more proximal. As the digestion of lipids is a highly efficient process, it is assumed that the majority of dietary lipids is hydrolysed and absorbed within the small intestine^(^ ^63^ ^)^. The presence of excess undigested lipids in faeces, referred to as steatorrhoea, is generally observed in individuals suffering from pancreatic or GI diseases (e.g. bile acids insufficiency)^(^ ^64^ ^)^ or in patients who have been administered lipase inhibitors such as the ones prescribed for weight management (e.g. tetrahydrolipstatin, generic drug name Orlistat)^(^ ^65^ ^)^. However, depending on the structure of the plant food ingested (e.g. cells/nutrients encapsulated by an indigestible barrier such as dietary fibre), a significant proportion of lipids and lipophilic nutrients such as carotenoids may pass from the ileum to the colon in healthy subjects^(^ ^66^ ^–^ ^69^ ^)^. Similarly, starch or protein that is resistant to digestion in the upper-GI tract (i.e. because it is physically inaccessible or resistant to enzymes) would be passed on to the colon, where the encapsulating fibre serves as a substrate for microbial fermentation. For this reason, by some current definitions, resistant starch is considered to be a type of dietary fibre^(^ ^4^ ^)^.

The plant food macronutrients and micronutrients that are not absorbed in the small intestine reach the colon, which is the main site of water and electrolyte uptake, and together with encapsulating fibre are potential substrates for the microbiota. SCFA, notably acetic, propionic and butyric acids, are produced in the colon from bacterial fermentation of dietary carbohydrates that have escaped digestion and absorption in the upper part of the GI tract^(^ ^70^ ^)^. SCFA represent a source of energy for the host and also improve the intestinal environment, which may have some significance in their potential role in the prevention and treatment of certain conditions including the metabolic syndrome and large bowel diseases^(^ ^71^ ^)^. Most of these SCFA are absorbed by colonic epithelial cells and either metabolised *in situ* or in the liver. However, not all carbohydrates that pass on to the colon are fully fermented, and may therefore be excreted in faeces^(^ ^72^ ^)^.

### Importance of food structure

Composition alone does not fully characterise a food product because other properties such as the food structure are highly relevant when interpreting data on food behaviour in the gut and its subsequent effects on postprandial metabolism. It is well established that not all nutrients contained within a food are necessarily available for digestion and absorption^(^ ^27^ ^)^. Nevertheless, consumers, scientists and healthcare providers often rely on the nutrient composition to assess the ‘health quality’ of a food. This follows from the fact that for most foods information about and understanding of food properties in the GI tract and nutrient bioaccessibility are severely limited^(^ ^28^ ^)^. This can lead to misconceptions about the impact of food on gut function, metabolism and long-term health. For instance, on the basis of nutrient composition information, nuts such as almonds contain what is generally considered to be an unhealthy amount of energy content and lipids. However, only a proportion of that lipid may actually be digested and absorbed^(^ ^73^ ^–^ ^75^ ^)^. Almonds may therefore be deemed healthier than what is expected from the list of nutrients declared on the food label. Similarly, knowledge of the dietary fibre content of a food does not provide any information on the integrity of the food matrix, the physico-chemical characteristics of the dietary fibre or the subsequent physiological effects (e.g. transit time and glycaemic response for starch-rich foods).

The impact of food structure/matrix has often been studied in the context of release and availability of micronutrients such as carotenoids and flavonoids^(^ ^69^ ^,^ ^76^ ^,^ ^77^ ^)^ but less so for macronutrients^(^ ^78^ ^)^. Thus, the digestion of protein, lipid and starch is relatively well understood from a molecular point of view (i.e. enzyme activity and digestion kinetics)^(^ ^32^ ^,^ ^63^ ^,^ ^79^ ^–^ ^82^ ^)^, but much less information exists on the physical and chemical transformations of complex food matrices in the different GI tract compartments and the resulting effects on nutrient bioaccessibility^(^ ^43^ ^,^ ^46^ ^,^ ^83^ ^)^. Understanding the structure and behaviour of plant foods during processing and digestion is of crucial importance for providing a deeper insight into digestive physiology and facilitating the design of food ingredients and products with optimal nutritional properties. During digestion, food morphology is altered as a result of enzyme activity and the physico-chemical conditions of the GI tract (e.g. pH, physical contractions due to peristalsis and GI secretions)^(^ ^30^ ^)^. Characterisation of food materials is an important step in performing mechanistic studies and should include not just a detailed chemical analysis of nutrients but a wider range of physical and chemical techniques including microscopy, thermal analysis (e.g. differential scanning calorimetry (DSC)), rheology and IR spectroscopy^(^ ^84^ ^,^ ^85^ ^)^. Such methods can provide valuable information on the structural organisation (e.g. cell shape, cell wall thickness, location and physical state of starch granules and oil bodies) and also on the properties of food components during digestion, including the molecular weight, composition and solubility of cell wall polysaccharides, and the viscosity and particle size of whole digesta.

The fate and characteristics of undigested food particulates are rarely mentioned in the literature, as it is frequently assumed that food particulates are fully disintegrated and dispersed in the aqueous phase of the digesta. Although many different sources and forms of dietary fibre have been tested in digestion studies^(^ ^86^ ^)^, often as isolated biopolymers (e.g. galactomannans, pectins and *β*-glucans) or as extracted particulates (e.g. wheat bran), studies of intact plant tissues, including the behaviour of cell walls, have received very little attention^(^ ^16^ ^,^ ^83^ ^)^. We have recently highlighted the importance of such studies by showing that particles of some plant food tissue (e.g. almonds, wheat and chickpeas) retain their structure and integrity at the later stage of digestion, so that the intact material appears at the terminal ileum^(^ ^7^ ^)^ and even in faeces^(^ ^66^ ^,^ ^68^ ^)^. However, our understanding of the structural characteristics of the wide range of foods in our diet and their behaviour during digestion is still limited.

With regard to nutrient organisation, processed foods such as mayonnaise and ice cream consist of easily available and digestible proteins and emulsified lipids. However, a considerable range of foods contain nutrients organised into complex structures – for instance, seeds and nuts. In plant foods, lipids are predominantly TAG assembled into oil bodies. These organelles are delimited by a monolayer of phospholipids in which proteins are embedded^(^ ^87^ ^)^. Although the behaviour of isolated oil bodies in the gastric and duodenal environments is beginning to be understood^(^ ^88^ ^,^ ^89^ ^)^, uncertainty remains regarding their fate in the GI tract when they are ingested as part of a food matrix. In particular, it is still unknown how their structure is affected by the mechanical and enzymic degradations occurring during digestion. This area of research should not be neglected as nuts and seeds are widely consumed worldwide and make an important contribution to the diet^(^ ^11^ ^)^.

Starch is a major source of dietary energy for consumers in the UK (contributing about 16–41 % of total food energy)^(^ ^90^ ^)^ and is usually the predominant source of dietary carbohydrate^(^ ^91^ ^)^. There are large variations in the duration and magnitude of the glycaemic and insulinaemic responses evoked by starch-rich foods, which have implications for risk factors of cardiometabolic diseases and long-term health maintenance^(^ ^9^ ^,^ ^10^ ^,^ ^92^ ^)^. A range of factors are known to influence starch digestibility, and therefore glycaemia, including starch granule structure, composition and botanical source^(^ ^32^ ^,^ ^93^ ^)^. Although some starches are consumed in their native/raw or partially gelatinised states (e.g. bananas, biscuits, respectively), the majority of it is consumed in a more disordered or gelatinised form. The native form has a highly ordered/crystalline structure, which usually exhibits limited susceptibility to amylolysis. When starch is subjected to hydrothermal processing, however, water ingress and heat causes it to become more amorphous and gelatinise, which is associated with an increase in its susceptibility to amylolysis^(^ ^93^ ^)^. If the starch is subsequently allowed to cool, re-crystallisation occurs, resulting in the formation of retrograded starch, which is less susceptible to amylolysis than gelatinised starch^(^ ^94^ ^)^. Temperature and water availability are key factors that influence the physical form of the starch, and manipulating these parameters during food processing can strongly influence its digestibility, and therefore glycaemic responses^(^ ^95^ ^)^. It has been suggested that when starch granules are entrapped in a food matrix or in plant cells, the heat, water or space required for granular swelling and gelatinisation is limited, resulting in partially swollen granules with a distorted shape^(^ ^60^ ^)^. Such distorted granules have been observed using microscopy in various hydrothermally processed foods^(^ ^60^ ^,^ ^96^ ^,^ ^97^ ^)^. Further evidence that the distorted shape may reflect incomplete gelatinisation is provided by DSC studies, which have demonstrated an increase in the extent of starch gelatinisation with increasing disruption of physical structure^(^ ^98^ ^,^ ^99^ ^)^. Thus, the food matrix could limit the inherent digestibility of starch, as partially gelatinised starch would be less susceptible to *α*-amylase than starch that has been fully gelatinised^(^ ^93^ ^,^ ^95^ ^)^.

## Dietary fibre sources, structures and properties

The main sources of dietary fibre are cereals, legumes such as beans and lentils, vegetables, fruits, nuts and seeds^(^ ^12^ ^,^ ^100^ ^–^ ^102^ ^)^. Fibre-rich foods, particularly unrefined grain products, are considered to form an important part of a balanced, healthy diet. The current recommended average fibre intakes for adults are 18 g/d in the UK and 21–38 g/d in the USA^(^ ^91^ ^)^. The lower recommended dietary fibre intake for UK is explained by the fact that this value is based only on the total amount of NSP, determined by the Englyst GC method^(^ ^103^ ^)^. The UK value for fibre as NSP corresponds to approximately 23–24 g/d of dietary fibre as determined by the gravimetric AOAC (Association Of Analytical Communities) method^(^ ^104^ ^)^, which is used for fibre analysis in the USA and elsewhere. The AOAC method is based on the definition from the Codex Alimentarius and measures not only NSP but also other components resistant to digestion, such as lignin, resistant starches and oligosaccharides, and hence the higher values. However, at present, neither the UK nor the US populations have met the recommended targets and the intake of dietary fibre still remains low.

### Physico-chemical properties of dietary fibre

Details about the chemical characteristics of dietary fibre, particularly the composition and structural architecture of the cell wall matrix can be found elsewhere^(^ ^31^ ^,^ ^100^ ^,^ ^101^ ^,^ ^105^ ^)^. In brief, cell walls of many edible plant tissues are made up of three main types of polysaccharides – cellulose, hemicelluloses and pectins. The combination of cellulose microfibrils and cross-linking hemicelluloses with an inter-penetrating pectin network provides strength and rigidity to the cell wall. In cellulose, a network of microfibrils formed by close packing of unbranched *β*-1,4-glucan chains, which are stabilised by intra- and inter-molecular hydrogen bonds, makes this polymer water insoluble and an extremely strong structure. Because of the linkage pattern of *β*-1,4-glucan chains, which are not hydrolysed by endogenous enzymes of the upper GI tract, and the tight packing of cellulose chains, cellulose is more resistant to mechanical, chemical and microbial degradation than any other polysaccharide found in cell walls^(^ ^1^ ^)^.

Cell walls of most edible tissues can be classified as type I or II on the basis of their polysaccharide compositions^(^ ^106^ ^)^. Dicotyledons (i.e. most fruits and vegetables), non-commelinid monocotyledons (e.g. asparagus and onion) and conifers have a type I primary cell walls. In addition to cellulose, the most abundant polysaccharides in type I cell walls are xyloglucan (a hemicellulose) and pectic polysaccharides. Type II primary cell walls are found in cereals and grasses and contain a high proportion of cellulose and the hemicelluloses arabinoxylan and mixed-linkage *β*-glucan, but they contain only negligible amounts of pectic polysaccharides and proteins. Despite numerous attempts to define structural models of plant cell walls, the details of their molecular architecture are not yet fully understood^(^ ^101^ ^)^, although the composition and structure of individual cell wall polysaccharides constituting many plants tissues are relatively well known^(^ ^5^ ^,^ ^107^ ^)^. However, notwithstanding the significant development in advanced technology (e.g. NMR) for studying cell walls^(^ ^108^ ^,^ ^109^ ^)^, there is still limited information describing the localised proportions of the cell wall polysaccharide constituents, including those of commonly consumed plant foods (e.g. nuts, legumes and cereals), because these can vary even around a single cell^(^ ^110^ ^)^. Moreover, information is also limited on the complex supramolecular assemblies of cell wall polymers, notably polymer–polymer interactions and phenolic cross-linking, but also on the size of pores or channels in the cell wall matrix, including the plasmodesmata that may have significant implications on the permeability of the cell walls to digestive enzymes^(^ ^111^ ^)^. It has been known for some time that cell wall polysaccharides ingested as individual polymers, which have been extracted and purified, are likely to behave differently in the GI tract compared with when they are part of intact cell walls or within a food matrix^(^ ^112^ ^)^. It is known, for example, that polysaccharides can interact with non-carbohydrate components of the cell wall and intra-cellular nutrients^(^ ^113^ ^,^ ^114^ ^)^. It has become apparent that the structure and organisation of the polysaccharides within the cell wall matrix play a significant role in their response to mechanical processing and digestion^(^ ^1^ ^,^ ^13^ ^)^.

It has become common practice to categorise different types of dietary fibre according to their extractability or dissolution in water (i.e. water soluble *v.* water insoluble fibre), values of which are often included in food composition tables and sometimes on food labels. However, water solubility of fibre is based on an *in vitro* method only and may not reflect the degree of solubility in the gut. Published values on total fibre content and the relative amounts of soluble and insoluble fibre encompass many plant-based materials ranging from fruits and vegetables and processed foods to purified extracts of NSP, some of which have been used as fibre supplements in foods and pharmaceutical preparations^(^ ^115^ ^)^. Examples of water-insoluble NSP include, not surprisingly, cellulose and chitin, and the water-soluble types include mixed-linkage *β*-glucans, pectins, galactomannans and also algal polysaccharides such as carrageenan and alginate. Many of the water-soluble polymers have been used as ‘model’ polysaccharides in mechanistic studies designed to elucidate the physiological actions of ‘soluble fibre’^(^ ^112^ ^)^. However, ‘model’ polysaccharides will be of less use when studying the properties of structurally intact plant cell walls, which are now known to act, *inter alia*, as a physical barrier to the bioaccessibility and digestion of intra-cellular nutrients^(^ ^15^ ^)^. Although the category of ‘insoluble fibre’ is used specifically to quantify insoluble components of the cell walls (e.g. cellulose), an intact cell wall matrix can also be defined as ‘insoluble’, even if some carbohydrate components of it may solubilise during food processing and digestion (e.g. mixed linkage *β*-glucan of oat cell walls)^(^ ^13^ ^)^. Current methods of chemical analysis of dietary fibre, however, are not able to characterise the physical state of cell walls or provide any useful information on properties relevant to their impact on gut function and postprandial metabolism, other than providing data on fibre content.

The physico-chemical mechanism(s) underlying the physiological effects of dietary fibre is still not well understood. However, we know already that a single, unified mechanism is unlikely to explain precisely how fibre influences macronutrient digestion and other gut functions, as well as how these complex processes are linked to postprandial metabolism^(^ ^3^ ^,^ ^116^ ^)^. A number of mechanisms are likely to be involved, but which of these predominates under certain conditions will depend on many factors including the polysaccharide composition and the physical state of the fibre source and whether or not the fibre-containing foods and drinks have been processed. Furthermore, the presence of confounding variables in foods (e.g. polyphenolics and lipid) that can also potentially influence gut function makes it difficult to delineate the physiological impact of fibre when part of a food matrix. This explains why the use of purified ‘model’ polysaccharides with defined physico-chemical characteristics have been particularly useful in mechanistic studies of dietary fibre^(^ ^115^ ^,^ ^117^ ^)^.

### Polysaccharide shape and solution properties

At the molecular level, the conformation or shape and chain packing of cell wall polysaccharides are critical factors that determine their behaviour in solution and their interactions with solvents, enzymes and other molecules^(^ ^117^ ^)^. Thus, most soluble forms of fibre, such as the leguminous galactomannans and xyloglucans, and the mixed-linkage *β*-glucans found in oats and barley, behave as fluctuating, disordered chains (i.e. ‘random coil’ polysaccharides) in solution. On the other hand, as mentioned above, a polysaccharide classified as insoluble fibre, either as a pure polymer or part of a cell wall, can adopt an ordered organisation where the glycan chains are packed tightly together into crystalline assemblies. Such highly ordered assemblies, as for instance found in cellulose microfibrils or raw starch granules, confer resistance to solubility and enzymic degradation.

Some polysaccharides can also form gel structures, which are hydrated, swollen polymer networks (e.g. pectins and alginates) that are cohesive and more solid-like and can support their own weight under the force of gravity. These hydrated networks (gels) exhibit such properties because within them co-exist sequences of the glycan chains that are ordered and disordered, which are characterised by the presence of ‘junction zones’ (stabilised by non-covalent bonding) and solubilised sequences, respectively. More complex heterogeneous tissue structures, including cell walls, of many raw and processed plant foods and also composite foods such as wheat bread could also be considered to be types of ‘hydrated networks’ during digestion in the GI tract. How such complex networks are deconstructed by physical force (peristalsis) and the plasticising effects of water and digestive fluids will have a significant bearing on the mechanisms of digestion.

The rheology of solutions containing pure polysaccharides is well understood and widely described in the literature^(^ ^13^ ^,^ ^115^ ^)^; it is well known, for example, that these solutions of soluble fibre exhibit shear-thinning (pseudoplastic) behaviour under increased mixing (shear rate) conditions. However, the rheological characterisation of digesta in the human GI tract presents a formidable challenge for many reasons, not least of which is gaining access to different sites of the gut for sampling. In contrast, pigs have been found to be a useful animal model for gaining access to different regions of the gut and studying such complex heterogeneous systems^(^ ^115^ ^,^ ^118^ ^,^ ^119^ ^)^. In physiological terms, the rate of shear or deformation is related to the degree of mixing of digesta caused by peristaltic contractions in the gut lumen, and this can have a major effect on, for instance, the rate of starch digestion^(^ ^120^ ^)^, although it should be borne in mind that the flow patterns *in vivo* are likely to be highly complex and variable. Digesta will be subjected to different shear rates in different locations of the GI tract and at different times. Other factors that are relevant to characterising the rheology of human digesta include the variable rates and extent of polysaccharide dissolution^(^ ^13^ ^,^ ^121^ ^)^ and variations in polysaccharide concentrations in different regions of the gut lumen due to fluctuations in water content. Furthermore, ‘insoluble’ particulates of rice starch or microcrystalline cellulose added to solutions of guar galactomannan are known to increase viscosity and modify rheological behaviour by becoming more rate dependent at low shear rates^(^ ^122^ ^)^. This behaviour has important implications for digesta viscosity as discussed below.

The molecular weight of individual polysaccharides in cell walls can also decrease within a fibre-rich food ingredient (i.e. cereal flours) owing to the presence of endogenous enzymes capable of hydrolysing polymer chains^(^ ^123^ ^,^ ^124^ ^)^. A number of studies have shown that mixed-linkage *β*-glucan can be depolymerised by *β*-glucanase during food preparation/processing, specifically cooking methods such as baking and also extensive extrusion^(^ ^125^ ^)^. The depolymerisation of *β*-glucan is reported to reduce the health benefits associated with the consumption of this fibre, as observed with bread, pasta, cookies and other products derived from oat and barley^(^ ^126^ ^,^ ^127^ ^)^. Moreover, processing and storage, including freezing, can also decrease the capacity of *β*-glucan to generate solution viscosity by reducing the extent of solubility of the polymer.

The physical properties of the digesta, particularly the size, shape and amount of insoluble particulates, and the proportion of solid and liquid phases will affect its viscosity, and therefore its flow behaviour, with or without the presence of soluble fibre. As a result of such changes in digesta rheology, the degree of mixing of enzymes and substrates, gastric empting rate, intestinal transit time and permeation by GI secretions, and thus the rate of digestion and absorption of nutrients, can be markedly affected^(^ ^56^ ^)^. One of the many complexities of measuring and interpreting digesta viscosity is the biphasic character of this non-steady state system, comprising solid particles dispersed in an aqueous phase. Each phase fluctuates significantly as digestion progresses – the digesta becomes diluted because digestive fluids are secreted in the upper part of the GI tract, whereas the volume of the aqueous phase diminishes throughout the small and large intestines as water is absorbed. Given that soluble fibres can affect viscosity only when they hydrate and form molecular solutions, as explained above, the viscosity of fluid digesta will depend on the proportion of dissolved polysaccharides. The physical state of soluble forms of fibre in foods that are consumed is thus likely to impact on digesta properties, their interaction with nutrients and enzymes and their susceptibility to microbial fermentation. In relation to the rheological properties of the digesta, the degree of viscosity generated by the soluble fibre in the proximal gut is highly dependent on the rate and extent of dissolution of polysaccharide chains and the molecular weight and concentration of dissolved/hydrated polymer. However, another important factor in influencing swelling and dissolution of soluble fibre is likely to be how it is consumed – for example, whether it is a part of low-moisture foods such as biscuits and crispbreads or more highly hydrated food systems such as porridge or comminuted fruit products (e.g. ‘smoothies’)^(^ ^117^ ^)^.

The effects of soluble fibre on limiting nutrient bioaccessibility/digestibility by increasing digesta viscosity in the gut lumen have been extensively studied in starch-rich plant foods, especially in relation to attenuating postprandial glycaemia^(^ ^128^ ^,^ ^129^ ^)^. An increase in viscosity in the GI tract following soluble fibre consumption also seems to be an important mechanism linked to improvements in lipid metabolism, particularly the lowering of fasting plasma cholesterol concentrations^(^ ^116^ ^,^ ^127^ ^,^ ^130^ ^)^. Some or all of these improvements in lipid and carbohydrate metabolism may occur as a result of a reduction in the rate of digestion and absorption of lipids and starch, respectively. Thus, soluble fibre may slow down macronutrient digestion by reducing the flow and mixing of digesta as a consequence of increased viscosity^(^ ^56^ ^)^. At low viscosity, the contents of the intestinal lumen may undergo rapid mixing generated by turbulent flow, whereas in laminar flow (caused by high viscosity) poor mixing conditions may occur^(^ ^131^ ^)^. In addition to decreasing the rate of nutrient digestion and mass transfer by inefficient mixing, viscous conditions may limit the diffusion of nutrients from the lumen to the mucosal epithelium.

Studies of the effects of the rheological behaviour of meals containing soluble fibre on gastric emptying have led to conflicting results. For instance, an early study in dogs showed that guar gum (galactomannan) induced a change in hydrodynamic factors by altering flow patterns in response to raised viscosity and also disrupted gastric sieving^(^ ^132^ ^)^. In a later study, Marciani *et al*.^(^ ^133^ ^)^ instructed human volunteers to ingest a viscous ‘meal’ of water containing different concentrations of locust bean gum, a galactomannan-rich soluble fibre, and then measured the gastric responses using MRI. The results showed that, regardless of the initial viscosity of the test meals, the viscosity of the solutions also increased in the stomach after 12 min of ingestion, but then decreased after 40 min probably because of dilution with saliva and gastric juices. This group also reported that the most viscous meal delayed gastric emptying to a greater extent than the meals of lower viscosity. In studies by other groups, an acceleration in gastric emptying was also reported following ingestion of a liquid meal enriched with pectin^(^ ^134^ ^)^, whereas Sanaka *et al*.^(^ ^135^ ^)^ showed the opposite effect. These discrepancies could be due to differences in study design, which could include differences in the methods used to measure gastric emptying and viscosity and the amount, type and hydration state of soluble fibre consumed.

## Specific mechanisms of dietary fibre action related to macronutrient digestion and absorption

### Bile salt metabolism

Water-soluble forms of dietary fibre have been reported to reduce fasting plasma cholesterol concentrations in humans (usually due to a lowering of the LDL fraction) via mechanisms that include modifications of bile salt metabolism^(^ ^136^ ^)^. Bile acids are synthesised in the liver from cholesterol and are conjugated to a molecule of either glycine or taurine to form bile salts^(^ ^137^ ^)^. Bile salts are stored in the gall bladder and then secreted into the duodenum where they facilitate solubilisation, and thus digestion and absorption of lipids in the GI tract. They achieve this by promoting the anchoring of lipase to the lipid–water interface of lipid droplets and removing lipolytic products that accumulate at the interface. Bile salts are subsequently re-absorbed in the ileum by specific Na-dependent transporters into the hepatic portal vein and returned to the liver for re-circulation (a process termed the enterohepatic circulation). Unabsorbed bile salts pass on to the colon where they may be deconjugated by bile salt hydrolases and then passively absorbed or excreted^(^ ^138^ ^)^.

Bile salts are natural surfactants, as they are amphiphilic molecules with an unusual structure composed of lipophilic and hydrophilic faces. The most abundant bile salts are cholate, deoxycholate and chenodeoxycholate conjugated with either glycine (75 %) or taurine (25 %)^(^ ^137^ ^)^. They position themselves at the lipid–water interface by projecting their hydrophilic face into the water and the hydrophobic one into the lipid phase^(^ ^139^ ^)^. The accumulation of bile salts at the interface of oil droplets reduces the surface tension of the droplets, and thereby facilitates the anchoring of the colipase and subsequently the pancreatic lipase^(^ ^140^ ^)^. In addition, bile salts have a ‘cleaning’ role as they remove from the interface other surface-active molecules such as proteins and lipolytic products; this action is attributed to their high surface tension^(^ ^137^ ^)^. Bile salts are also involved in solubilising the products of lipolysis and integrating them into micelles.

Animal and human studies have shown that the ingestion of soluble types of dietary fibre, notably oat *β*-glucan and guar gum, elicited a reduction in plasma cholesterol levels and an increase in bile acid excretion^(^ ^141^ ^–^ ^144^ ^)^. Thus, soluble fibre is thought to have an impact on bile salt recycling plus mixing and transport of mixed micelles^(^ ^136^ ^)^. It has been suggested that soluble forms of fibre exert their plasma cholesterol-lowering effect by direct ‘binding’ of polysaccharides to bile salts, although the type of binding involved here is unclear^(^ ^136^ ^,^ ^145^ ^–^ ^147^ ^)^. A more likely explanation may be that there is a temporary entrapment of the bile salts within the viscous network of entangled polysaccharide chains^(^ ^148^ ^–^ ^151^ ^)^. Soluble fibre may therefore impair convective mixing in the gut lumen and limit the effectiveness of the emulsification process and mass transfer of lipolytic products to the mucosal surface^(^ ^136^ ^)^. The bile salts that are unabsorbed from the terminal ileum or the large intestine will then be either metabolised by colonic fermentation and/or excreted in the faeces. The depletion of the native bile acid pool by soluble fibre, through metabolism and/or excretion, requires catabolism of cholesterol in the hepatocytes to replenish this pool. However, our understanding of the possible molecular interactions between bile salts and/or micelles and dietary fibre is still somewhat limited^(^ ^151^ ^)^. Moreover, detailed information of how different chemical and physical forms of dietary fibre, for example, solution behaviour of soluble fibre *v.* insoluble matrices of cell walls, can affect such interactions and other digestive processes is still lacking.

### Interactions with enzymes and/or substrates

The presence of water-soluble dietary fibre in the digesta could minimise the interactions between enzymes and substrates because of the increase in viscosity and subsequent reduced mixing in the GI tract. In addition, dietary fibre present as solubilised polysaccharide chains may reduce macronutrient hydrolysis by direct binding to digestive enzymes and/or by physical interaction (binding) with hydrophilic substrate surfaces. Indeed, the results of previous studies have suggested that guar galactomannan inhibits the rate of starch digestion by one or both of these mechanisms^(^ ^18^ ^,^ ^82^ ^)^. In an *in vitro* enzyme kinetic study, guar galactomannan was observed to act as a non-competitive inhibitor of amylase, by binding to the enzyme but not specifically to the active site^(^ ^80^ ^)^. In an earlier study of pigs that ingested wheat bread containing guar gum, starch granules appeared coated with a layer of galactomannan. This polymer layer may limit the access of *α*-amylase to starch as also observed more recently^(^ ^152^ ^)^. The reduction in starch hydrolysis in the presence of soluble dietary fibre can also be caused by a restriction in water availability necessary for the swelling and gelatinisation of starch granules^(^ ^153^ ^)^. More recently, it has been revealed that cellulose, an insoluble form of fibre, and purified wheat bran, which contains cellulose, inhibit starch hydrolysis^(^ ^154^ ^)^. Cellulose seems to inhibit the activity of *α*-amylase by non-specific binding involving a mixed-type inhibition mechanism.

Limited *in vivo* data are available on the effect of dietary fibre on lipase activity. An early study performed on subjects with pancreatic insufficiency revealed a decrease in lipase activity following ingestion of pectin and wheat bran^(^ ^155^ ^)^. Lipid digestion may be influenced by dietary fibre through the formation of a coating around lipid droplets in an analogous process to that observed with starch. *In vitro* studies performed on chitosan (a water-soluble polysaccharide of d-glucosamine units formed by deacetylation of chitin) showed that it formed a polymer coat on the surface of lipid droplets, thus preventing the adsorption of the lipase/colipase complex to the interface^(^ ^156^ ^,^ ^157^ ^)^. In other studies, emulsification and hydrolysis of TAG in the gastric environment were also reported to be decreased in the presence of guar gum and apple pectins^(^ ^158^ ^)^. However, conflicting results are found in the literature regarding the effect of pectic polysaccharides on lipid digestion. Some studies have shown that pectins inhibit lipase activity^(^ ^157^ ^,^ ^159^ ^,^ ^160^ ^)^ and promote aggregation of lipid droplets^(^ ^161^ ^)^. In contrast, other research groups have reported that pectins have little impact on lipid digestibility^(^ ^162^ ^,^ ^163^ ^)^. The discrepancies observed in these physiological outcomes are likely to be due to the variations in the structure and properties of the pectins studied (e.g. degree of esterification, molecular weight and concentration), as well as the experimental conditions (e.g. pH and lipolytic substrate). These parameters seem to be major determinants of the behaviour of polysaccharides *in vivo* (see above). Finally, inhibitory compounds such as phenolic compounds or peptides initially present in the food matrix or generated during food processing (e.g. roasting of almonds), could be released during digestion, potentially reducing the rate of lipolysis^(^ ^164^ ^,^ ^165^ ^)^.

### Encapsulation of nutrients within the food matrix

The structure and properties of the cell wall play an important role in regulating the release/availability of micronutrients and macronutrients (e.g. carotenoids, lipid and starch) from plants foods during mastication and digestion^(^ ^6^ ^,^ ^31^ ^,^ ^39^ ^,^ ^68^ ^,^ ^69^ ^,^ ^166^ ^)^. Generally, in order to be digested, the nutrients have to be released from the food matrix, and thereby become available for digestion and absorption at the appropriate site of the GI tract. However, a significant proportion of cell walls may remain intact even after mastication and other phases of the digestion process and, as a result, may decrease the rate and extent of nutrient digestion and the postprandial metabolic response. Cell walls remain resistant to digestion in the upper GI tract of humans, because endogenous enzyme secretions (e.g. amylases) are unable to hydrolyse polysaccharide components of the plant cell walls^(^ ^167^ ^)^. The digestive enzymes acting on available carbohydrates in humans (i.e. *α*-amylase and disaccharidases) can only hydrolyse *α*-1,4- and *α*-1,6-glucan linkages of starch^(^ ^1^ ^)^.

The structure and behaviour of individual cell wall polysaccharides and macroproperties of the cell wall matrix have a significant bearing on how plant foods disassemble and release nutrients during digestion. In earlier studies dating from the 1990s, foods containing intact macroparticles (e.g. pumpernickel-style bread or spaghetti) have been shown to elicit a significantly lower glycaemic response than a de-structured equivalent (e.g. wholemeal bread or chopped spaghetti)^(^ ^168^ ^,^ ^169^ ^)^. An investigation performed in human participants with ileostomies who received different physical forms of carrot (raw grated or cooked mashed) revealed that the loss of cell wall integrity (as represented by ruptured cells and cell wall swelling) led to an increase in carotene bioaccessibility^(^ ^69^ ^)^. Another study, carried out on peanuts, indicated that the degree of comminution of the food structure altered the quantity of fat absorbed^(^ ^170^ ^)^, and these findings were later confirmed^(^ ^171^ ^)^. More recently, in an ileostomy study, intact particles of ingested durum wheat endosperm were identified at the terminal ileum following up to 10 h of gut residence^(^ ^7^ ^)^. In these digested endosperm macroparticles that had been cooked and prepared as porridge, the plant cell walls appeared intact. However, much of the intra-cellular starch was found to be digested, showing a progressive loss of starch from the particle periphery towards the core, and therefore suggesting a gradual penetration of amylase through permeable endosperm cell walls. In an *in vitro* digestion study, it was reported that after gastric and duodenal digestions of masticated whole almonds, a large proportion of lipids (approximately 68 %) remained enclosed within the particles^(^ ^57^ ^)^. The overall structure of tissues during gastric digestion appeared to be relatively unaffected, especially for large particles. Duodenal digestibility experiments showed that there was an inverse relationship between particle size of almonds and the rate and extent of digestion; however, the almond samples with the lowest level of lipid digestion were the laboratory-separated almond cells (approximately 35 μm diameter) with intact cell walls^(^ ^16^ ^)^. As lipids provide most of the energy obtained from almonds (approximately 50 % of their content), a significant discrepancy would be expected to arise between their estimated energy content listed on a food label and the actual metabolisable energy available from digestion, absorption and fermentation. Indeed, it has been recently revealed that the commonly used Atwater factors markedly overestimates the available energy found in almonds^(^ ^75^ ^)^, walnuts^(^ ^74^ ^)^ and pistachios^(^ ^73^ ^)^ by about 32, 27 and 5 %, respectively, compared with experimental measurements of metabolisable energy. This highlights a major limitation of current nutrition labelling systems, and is likely to apply not just to almonds and other tree nuts but also to other food matrices in which a proportion of the nutrients is not bioaccessible. Intact cell walls therefore play an important role in limiting and/or delaying nutrient bioaccessibility and enzymic hydrolysis as they physically encapsulate the intra-cellular contents.

The different chemical and physical processes occurring during digestion appear to be unable to disturb the resilient cell wall matrix of hard, brittle plant foods such as almonds^(^ ^16^ ^)^. Nutrient digestibility is, however, variable between plant foods as cell wall properties can differ greatly between plant species and even within the same plant. Among cereals and legumes, chickpeas have cell walls that are thicker and less permeable to digestive enzymes than durum wheat cell walls that are relatively thin^(^ ^36^ ^)^. Chickpeas cells have the capacity to separate, whereas durum wheat cells do not separate after hydrothermal processing. As a result, starch encapsulated within chickpea cells was found to escape digestion entirely, whereas starch encapsulated within durum wheat cells was seen to be digested, although at a relatively slow rate. In starch-rich foods, it has been suggested that cell walls may limit starch digestibility via three mechanisms: first, the difficulty for amylase to permeate through the cell wall; second, the cell walls may limit starch gelatinisation during cooking; and, third, the binding of *α*-amylase by cellulose and potentially other cell wall components^(^ ^154^ ^)^. The capacity of cell walls to limit digestibility is therefore dependent on the extent to which cellular integrity is preserved during processing, mastication and digestive transit, which, in turn, vary between plant species. Pulses, for instance, tend to retain their cellular integrity during cooking and digestion, and food materials containing intact cells have been shown to have a reduced susceptibility to starch hydrolysis and evoke lower glycaemic responses compared with de-structured equivalents (i.e. beans or pea flour)^(^ ^61^ ^,^ ^83^ ^)^. In contrast, during hydrothermal processing of some varieties of potatoes, the cells can rupture, and thus starch is able to gelatinise more fully and is more susceptible therefore to hydrolysis by *α*-amylase^(^ ^172^ ^,^ ^173^ ^)^.

Nutrients released from plant tissues in the oral cavity and the subsequent sites of the GI tract may trigger neuronal and humoral signals that have an impact on digestive processes such as gastric emptying, peristaltic contractions and the ileal brake^(^ ^62^ ^,^ ^174^ ^)^. For instance, the release of gut hormones (e.g. glucagon-like peptide-1 (GLP-1), peptide YY (PYY), cholecystokinin (CCK) and gastric inhibitory polypeptide (GIP)) can be triggered by specific nutrient-sensing enteroendocrine cells that are present throughout the GI tract or through more complex neuro-endocrine signalling pathways. Thus, foods in which nutrients are encapsulated within the food matrix may not be able to trigger hormone signals in the small intestine compared with foods in which the nutrients are bioaccessible and more likely to be ‘detectable’.

### Microbial degradation of dietary fibre

The gut microbiota markedly differs in diversity and quantity between humans, even among healthy individuals^(^ ^175^ ^)^, and varies significantly with age and in different anatomical regions of the GI tract^(^ ^176^ ^)^. Human microbiota is dominated, in healthy adults, by two phyla – Bacteroidetes and Firmicutes – whereas Actinobacteria, Proteobacteria and Verrucomicrobia are also frequently found but in minor proportions^(^ ^177^ ^)^. Although many bacteria share common functions (i.e. carbohydrate and amino acid metabolism), certain activities (e.g. vitamin catabolism and nutrient transport) are restricted to some species and/or strains^(^ ^178^ ^)^. A close relationship, posing a balance between benefit and harm, exists between the host and the gut microbial communities. The microbiota is sensitive to external factors, with diet (both short and long term) being likely to have an important effect^(^ ^176^ ^)^. Different types and amounts of dietary fibre have been shown to have a variable impact on faecal microbial composition, but the modifications observed are not universal and rely on the initial gut microbiota. Bacterial species with a greater degree of nutritional diversity or flexibility appeared more resilient to dietary changes.

Undegraded cell wall material from the small intestine reaches the colon where water-soluble polysaccharide components of the cell wall are susceptible to rapid fermentation by the microbiota producing gases (i.e. hydrogen, methane and carbon dioxide) and SCFA, whereas most of the celluloses and less-soluble hemicelluloses, as well as cross-linked pectin, tend to be fermented more slowly and remain more intact^(^ ^100^ ^)^. Some cell walls from almond seeds were also found to be apparently undisturbed in human faeces despite detection of bacterial activity^(^ ^66^ ^)^. As previously explained, the differences in digestibility observed among cell wall types are likely to be due not only to their composition but also to the inter-polymer cross-links between the polymers, particularly lignocellulose-rich cell walls, which have very limited hydration capacity^(^ ^179^ ^)^. Non-digestible oligosaccharides and resistant starch can also escape digestion in the upper part of the GI tract and behave similarly to cell wall forms of dietary fibre and promote the growth of bacteria and production of SCFA^(^ ^180^ ^)^. These can normally represent a significant proportion of the total dietary fibre intake, particularly in diets rich in legumes or cereals containing raw, retrograded or entrapped starch.

The composition of the gut microbiota and its interactions with dietary fibre are contingent on the physico-chemical attributes of the different forms of fibre, which are known to influence their susceptibility to microbial fermentation. In addition, dietary fibre supplements incorporated into foods, for example, inulin, psyllium and arabinoxylan, are expected to be more readily available. These may well be degraded differently and at faster rates compared with cell wall material consumed as part of a plant-based diet (e.g. nuts and seeds, beans or wholegrain bread). Details about the degradation of dietary fibres in the colon and its physiological effects have been presented extensively in the literature and interested readers are referred to these articles^(^ ^181^ ^,^ ^182^ ^)^.

### Interaction with the mucus layer

Mucus gel layers cover the GI tract from the stomach to the colon^(^ ^183^ ^)^. Mucus is secreted by epithelial cells and is composed of water, biopolymers (mainly the glycoprotein mucin), bacteria and cell debris^(^ ^184^ ^)^. There are two mucus layers, the firmly adherent and the loosely adherent layers^(^ ^185^ ^)^. These mucus layers vary in thickness throughout the GI tract and provide a protective barrier against auto-digestion and pathogens. Mucins are a heterogeneous group of molecules with molecular weights ranging from 0·5 to 20 MDa^(^ ^186^ ^)^ and can aggregate and form gel-like structures owing to their complex colloidal behaviour. Indeed, mucins exhibit electrostatic, hydrophobic and hydrogen-bonding interactions, which impact on the mucus gel properties. The diffusion of molecules through the mucus gel is affected by the presence of mucins (mucoadhesivity), which are also a potential growth substrate for intestinal bacteria.

Dietary fibre may interact with the intestinal mucus layer, particularly with the mucins, and affect the thickness of the layers, and thereby nutrient absorption^(^ ^187^ ^)^. Oligosaccharides from alginate have been reported to decrease the viscosity of the mucus layer by disturbing the cross-linking mucin network^(^ ^188^ ^)^. In rats, the opposite effect was observed, where consumption of different types of fibre, including guar galactomannan and citrus fibre, which is rich in pectic polysaccharide, increased mucin production, thus creating a barrier to absorption of hydrolysis products^(^ ^189^ ^,^ ^190^ ^)^. This is likely to have been caused by the abrasive effects of the fibre, removing mucus and increasing luminal concentrations, and may include goblet cell proliferation induced by increased intra-luminal pressure. The exact impact of dietary fibre on the mucus layer is an area that warrants further research, given the potential effects on nutrient absorption.

### Conclusions

There is a large body of evidence to show that dietary fibre can significantly decrease the rate and extent of nutrient bioaccessibility, digestion and absorption in the upper GI tract, although the degree to which this occurs is variable and highly dependent on the structure and properties of the fibre source. Recent results from *in vitro* and *in vivo* studies highlight the significant role played by structurally intact cell walls of edible plants in impeding the digestion of intra-cellular starch and lipids. This cell wall barrier mechanism not only involves a restriction of enzyme–substrate interactions but also, in the case of starch granules, a decrease in the capacity of granules to swell and gelatinise, and therefore their susceptibility to amylolysis. In our opinion, this evidence represents a paradigm shift in understanding of how fibre affects the time course of digestion and absorption, as the prevailing view until recently has been that only soluble fibre impedes the digestion process itself.

The use of chemical analysis of dietary fibre alone for characterising the physiological activity of fibre in plant foods is extremely limited, especially when used to interpret mechanistic data on the behaviour of cell wall matrices or individual NSP. Moreover, values of dietary fibre content in foods, including *in vitro* estimates of ‘soluble’ and ‘insoluble’ fibre fractions, are of limited use to consumers beyond that of identifying foods that are low and high in fibre. One of the many challenges for researchers in the future will be to improve our understanding of the physico-chemical properties of dietary fibre at different sites of the GI tract and how this impacts on gut function and postprandial metabolism. This will also need to include a detailed characterisation of soluble fibre in the gut, including dissolution kinetics and molecular weight measurements, in conjunction with *in vitro* digestion assays that can model the behaviour of digesta. At a higher-length scale, knowledge of the mechanical properties (e.g. fragmentation) and porosity of cell wall matrices would add greatly to our understanding of the important physiological role(s) of dietary fibre during digestion.
